# Pulmonary Involvement in SARS-CoV-2 Infection Estimates Myocardial Injury Risk

**DOI:** 10.3390/medicina58101436

**Published:** 2022-10-11

**Authors:** Eduard Dumea, Mihai Lazar, Ecaterina Constanta Barbu, Cristina Emilia Chitu, Daniela Adriana Ion

**Affiliations:** 1 Faculty of Medicine, University of Medicine and Pharmacy Carol Davila, No. 37, Dionisie Lupu Street, Sector 2, 020021 Bucharest, Romania; 2 National Institute for Infectious Diseases “Prof. Dr.Matei Bals”, No. 1, Calistrat Grozovici Street, Sector 2, 021105 Bucharest, Romania

**Keywords:** cardiac injury, myocardial injury, SARS-CoV-2 infection, COVID-19 complications, COVID-19 risk factors

## Abstract

*Background and Objectives*: Severe acute respiratory syndrome coronavirus 2 (SARS-CoV-2) infection represents a pathology with primary pulmonary involvement and multisystemic impact, including cardiovascular injuries. The present study aimed to assess the value of clinical, biochemical, and imaging factors in COVID-19 patients in determining the severity of myocardial involvement, and to create a model that can be used toevaluate myocardial injury risk based on clinical, biochemical and imaging factors. *Materials and Methods*: We performed an observational cohort study on 150 consecutive patients, evaluating their age, sex, hospitalization period, peripheral oxygen saturation (SpO_2_) in ambient air, systolic and diastolic blood pressure, heart rate, respiratory rate, biochemical markers of cardiac dysfunction (TnI, and NT-proBNP), inflammatory markers (C reactive protein (CRP), fibrinogen, serum ferritin, interleukin-6 (IL-6), tumor necrosis factor alpha (TNFα)), D-dimers, lactate dehydrogenase (LDH), myoglobin and radio-imaging parameters. All patients underwent computerized tomography chest scan in the first two days following admission. *Results*: We observed elevated heart and respiratory rates, higher systolic blood pressure, and a lower diastolic blood pressure in the patients with cardiac injury; significant differences between groups were registered in TnI, NT-proBNP, LDH, CRP, and D-dimers. For the radiological parameters, we found proportional correlations with the myocardial injury for the severity of lung disease, number of pulmonary segments with alveolar consolidation, number of pulmonary lobes with pneumonia, crazy paving pattern, type of lung involvement, the extent of fibroatelectatic lesions and the mediastinal adenopathies. *Conclusions*: Myocardial injury occurred in 12% of patients in the study group. Ground glass opacities, interstitial interlobular septal thickening (crazy paving pattern), fibroatelectasic lesions and alveolar consolidations on CT scan were correlated with myocardial injury. Routine lung sectional imaging along with non-specific biomarkers (LDH, D-dimers, and CRP) can be further valuable in the characterization of the disease burden, thus impacting patient care.

## 1. Introduction

Severe acute respiratory syndrome coronavirus 2 (SARS-CoV-2) infection exerts a primary pulmonary involvement; however, it also affects the cardiovascular system, and has an important impact on patient outcomes [1]. Although respiratory failure is considered the main cause of death in COVID-19, cardiovascular symptoms such as palpitations, dyspnea, fatigue, and chest pain are frequently present both in the subacute and chronic stages of the disease [2]. The most reported cardiovascular complications in COVID-19 are represented by acute myocardial injury, myocarditis, pericarditis, heart failure, and deep venous thrombosis [3,4]. The elderly population with comorbidities presents an increased risk not only for COVID-19, but also for its complications [3]. While few papers have been published regarding the imaging diagnosis and cardiovascular manifestations of COVID-19 [5,6], they have been mainly based on magnetic resonance depiction of myocardial involvement, and to our knowledge, no study has tried to match pulmonary involvement patterns with biochemically proven cardiac injury.

The pulmonary involvement patterns found in patients with COVID-19 are represented by ground-glass opacities with or without thickened intralobular septa, alveolar consolidation, and fibroatelectatic lesions. Since the outbreak of the pandemic, the ground-glass opacities have been described as hazy areas with increased density in lung parenchyma, which do not obscure bronchial or vascular structures, and it has been hypothesized that they occur due to partial filling of airspaces and initial interstitial thickening [7]. Post-mortem biopsies have revealed pulmonary edema and hyaline membrane formation in COVID-19 patients, most probably due to the inflammation setting [8]. Alveolar consolidation refers to complete air replacement in the alveoli that obscures the margins of bronchial and vascular walls, and it is believed to suggest a progression of the ground glass opacities as well as the disease [9]. Fibroatelectatic lesions may occur in persistent lung involvement and incomplete remission of the alvelolar consolidation.

The present study aimed to assess the value of clinical, biochemical and imaging factors (computerized tomography of the thoracic segment) in COVID-19 patients in determining the severity of myocardial involvement at admission, and to create a model that can determine the risk of myocardial injury based on clinical, biochemical and imaging factors at admission. 

## 2. Materials and Methods

We performed an observational cohort study on 150 consecutive patients admitted to Department 2 of the National Institute of Infectious Diseases Prof. Dr. Matei Bals, Romania, between March 2021 and June 2021, dividing them into two groups: Group A (18 patients with biochemical markers of myocardial injury) and Group B (132 patients without biochemical markers of myocardial injury). 

For the sample size, we considered a confidence level of 95%, a margin of error of 5% and a population proportion of 5.4% (representing the prevalence of COVID-19 in general population in our country at the time of the study [10]), and calculated the minimum sample size using following formula: n = [z^2^ × p^x (1 − p^)]/ɛ^2^] (n = sample size, z = z score, p^ = population proportion, ɛ = margin of error). The resulting minimum population of patients was 79. 

**Figure 1:**
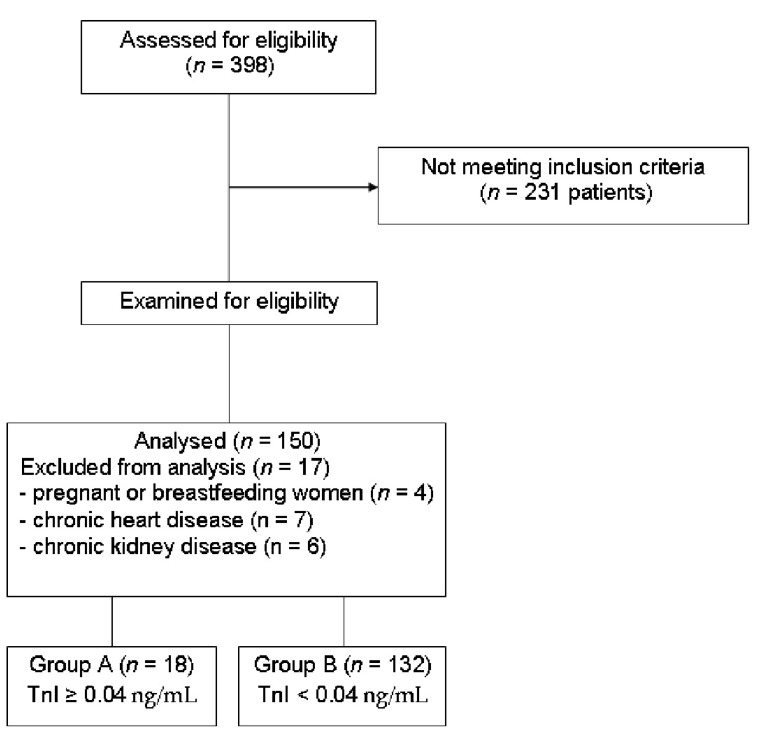
The flow diagram of the study describing recruitment, inclusions and exclusions in the study. Abbreviations: TnI-troponin I.

We considered an elevated serum value for troponin I (TnI) greater than 0.04 ng/mL as a biochemical marker of myocardial injury. All the data in this study were collected upon admission of patients, and the following inclusion criteria were used: age over 18 years, confirmed diagnosis of COVID-19 by Real Time-Polymerase Chain Reaction (RT-PCR), and chest computerized tomography (CT) on admission or within the first two days following admission. The exclusion criteria included age under 18 years, pregnant or breastfeeding women, patients with readily myocardial injury before admission orinfection with COVID-19, chronic heart disease, and chronic kidney disease (Figure 1). At the time of enrolment in the study no patient was on any cardiotoxic medication.

### 2.1. Demographic, Clinical and Biologic Parameters

The following data were recorded for each participant: age, sex, hospitalization period, peripheral oxygen saturation (SpO_2_) in ambient air, systolic and diastolic blood pressure, heart rate, respiratory rate, biochemical markers of cardiac dysfunction (TnI andNT-proBNP), inflammatory markers (C reactive protein (CRP), fibrinogen, serum ferritin, interleukin-6 (IL-6), tumor necrosis factor alpha (TNFα)), D-dimers, lactate dehydrogenase (LDH), myoglobin and radio-imaging parameters, as described below.

### 2.2. Radio-Imaging Parameters

Chest CT acquisitions were non-enhanced, with a tube voltage of 120 kV, in the automatic tube current adjustment mode in the craniocaudal direction, with a field of view of 300 mm, extending from the lung apices to below the diaphragm, with a 3 mm slice thickness, pitch of 1.5, and lung and soft tissue kernel reconstruction on a single breath-hold inspiration, by using a Somatom Sensation 64, Siemens AG, Erlangen, Germany. 

The imaging characteristics were categorized as follows:

- Severity of lung disease:Grade 0 (no pulmonary involvement), Grade 1 (mild pulmonary involvement: 1–5 pulmonary segments with pneumonia), Grade 2 (medium pulmonary involvement: 6–11 pulmonary segments with pneumonia), Grade 3 (severe pulmonary involvement: 12–19 pulmonary segments with pneumonia)
- Number of pulmonary segments with interstitial lung involvement (from 0 to 19)
- Number of pulmonary segments with alveolar consolidation (from 0 to 19)
- Number of pulmonary lobes with pneumonia (including both interstitial involvement and alveolar consolidation);
- Type of lung involvement: unilateral (1) or bilateral (2)
- Crazy paving pattern (interlobular septal thickening):Category 1 (changes are present in less than a third of pulmonary segments with ground glass lesions), Category 2 (changes are present in one-third to two-thirds of pulmonary segments with ground glass lesions) Cnd category 3 (changes are present in more than two-thirds of pulmonary segments with ground glass lesions)
- Vascular ectasia—Category 0 (when no vascular ectasia is noted), Category 1 (when less than half of the pulmonary segments with ground glass lesions show superimposed vascular ectasia), and Category 2 (when more than half of the pulmonary segments with ground glass lesions show superimposed vascular ectasia)
- Fibro-atelectatic lesions: Grade 0 (no pulmonary sign of fibroatelectatic lesions), Grade 1 (associated with mild pulmonary involvement: 1–5 pulmonary segments with fibroatelectatic lesions), Grade 2 (associated with medium pulmonary involvement: 6–11 pulmonary segments with fibroatelectatic lesions), and Grade 3 (associated with severe pulmonary involvement: 12–19 pulmonary segments with fibroatelectatic lesions);
- Mediastinal adenopathy: presence or absence of adenopathy measuring more than 1 cm in the short axis
- Pericardial effusion (present or absent)
- Pleural effusion (present or absent, whether unilateral or bilateral)

Two radiologists (ED and ML) with 5 and 20 years of experience in thoracic radiology, respectively, assessed the CT scans in a consensus reading for image quality and parenchymal changes, and both radiologists were blinded towards the identity of the patients as well as their demographic, clinical, and biological parameters.

### 2.3. Statistic Evaluation

Statistical Package for Social Sciences (SPSS version 25, IBM Corp., Armonk, NY, USA) was used for the statistical analysis. Patient data are presented as medians and quartiles (Q1, Q3) for continuous variables and as percentages for the categorical variables. 

The Spearman’s correlation was used to establish the independent variable that showed the highest correlation with the cardiac injury. 

We evaluated the impact of all parameters on cardiac injury using binary logistic regression univariate analyses with the registered data as the independent variables and cardiac injury as the dependent variable, followed by multivariable analysis.

The odds ratio (OR) for the variables with statistically significant variation was calculated using logistic regression. 

All the variables with significant association with the cardiac injury (*p* < 0.05) were grouped into demographic, clinical, biological, and imaging parameters. We evaluated the correlations between the recorded parameters to avoid collinearity (R^2^ > 0.7; *p* < 0.0001), and where any such pairs were found, the predictor with the lowest *p*-value was included in theanalysis and the other was ignored. 

For the multivariable logistic regression, we performed a backward elimination method: the independent variables were excluded based on the results of the Wald test for the individual parameters obtained during logistic regression analysis, and the least significant effect with *p* < 0.2 was removed from the prognostic model. The statistical significance of the logistic regression was estimated using the Omnibus test of model coefficients.

This study was performed in line with the principles of the Declaration of Helsinki and approved by the local ethical committee (C10218/15.09.2021).

## 3. Results

Group A, which included patients with biochemical evidence of myocardial injury as defined above, consisted of 18 participants (12%)—10 men (55.5%) and 8 women(44.5%)—while Group B, which included patients without biochemical evidence of myocardial injury, consisted of 132 participants (88%)—65 men (49.2%) and 67 women (50.8%). The sex ratios (male: female) in Groups A and B were 1.25:1 and 0.97:1, respectively, while the median age in Group A was slightly higher than that in Group B (58 years vs. 54 years).

All patients were admitted with the diagnosis of SARS-CoV-2 infection. The median duration from the first positive PCR test was 2 days for patients in both groups, with interquartile ranges (IQR), Q1 and Q3, of 1 (1.75; 3) and 2 (1; 3) for the patients in Groups A and B, respectively. We recorded prior SARS-CoV-2 infections in one patient in Group A (5.5%) and three patients in Group B (2.27%). Vaccination status was positive for two patients in Group A (11.1%) and 24 patients in group B (18.8%). The comorbidities of patients in both groups are presented in Table 1, with overweight/obesity representing the most frequent comorbidity in both groups and a higher percentage of overweight patients in Group B. Smoking status and dyslipidemia were found in similar proportions in both groups, while systemic arterial hypertension was more frequent in Group A (33.3%).

Elevated heart and respiratory rates, higher systolic blood pressure, and lower diastolic blood pressure were recorded in patients with cardiac injury. Significant differences in TnI, NT-proBNP, LDH, CRP, and D-dimerswere observed between the two groups. Furthermore, cardiac disfunction, illustrated by elevated NT-proBNP, was observed in six patients (33.3%) from Group A and 24 patients (18.18%) from Group B. Meanwhile, most of the inflammatory markers (except CRP) did not reach a significant *p*-value. The median levels ofserum ferritin, fibrinogen, and IL-6 were higher in Group A than in Group B; however, similar TNFα values were recorded in both groups (Table 2).

From CT scan results at admission, there were statistically significant differences in the severity of lung disease, number of pulmonary lobes with pneumonia, presence of crazy paving aspect, extent of fibroatelectatic lesions, and the presence of mediastinal adenopathy between participants in the two groups. The highest OR was observed in the case of mediastinal adenopathy (2.8), followed by the extent of fibroatelectatic lesions (2.4), crazy paving aspect (2.2), severity of lung disease (2.1), and the number of pulmonary lobes with pneumonia (1.6) (Table 3).

An increase ofone degree in the severity of lung disease increased the risk of myocardial injury by 118%; an increase ofone lobe in the number of pulmonary lobes with pneumonia increased the risk of myocardial injury by 61.8%; an increase ofone degree in the crazy paving aspect increased the risk of myocardial injury by 125%; an increase ofonedegree in the extent of fibro-atelectatic lesions increased the risk of myocardial injury by 144%; the presence of mediastinal adenopathies increases the risk of myocardial injury by 187% in the patients with SARS-CoV-2 pneumonia.

D-dimers showed the highest correlation with myocardial injury (Spearman’s rho = 0.322), followed by NT-proBNP, CRP, and LDH (Table 4).

For the radiological parameters, a proportional correlation with myocardial injury was observed with the severity of lung disease, number of pulmonary segments with alveolar consolidation, number of pulmonary lobes with pneumonia, crazy paving pattern, type of lung involvement, extent of fibroatelectatic lesions and the mediastinal adenopathies.

LDH, D-dimer levels, extent of fibroatelectatic lesions, and presence of mediastinal adenopathieswere found to be valuable in predicting myocardial injury in COVID-19 patients, as shown in the multivariable logistic regression model depicted in Table 5. In determining whether a cardiac injury may be anticipated based only on radio-imaging and inflammatory parameters, no cardiac markers (TnI or NT-proBNP) were included in the multivariable regression model, which had an overall prediction percentage of 87.4% and *p*-value < 0.001.

Based on the data in Table 4, we can also calculate the probability of myocardial injury using the following formula: EXP (Constant + 0.004 × LDH + 0.001 × D-dimers + 0.881 × Extent of fibroatelectasis lesions + 1.496 × Mediastinal adenopathies)/[1 + EXP (Constant + 0.004 × LDH + 0.001 × D-dimers + 0.881 × Extent of fibroatelectasis lesions + 1.046 × Mediastinal adenopathies)].

## 4. Discussion

From this study, the extent of interstitial pneumonia and superimposed interlobular septal thickening (“crazy paving pattern”), vascular ectasia, pulmonary lobes with pneumonia, pulmonary segments with alveolar consolidation, and pleural effusion were observed in patients with myocardial injury.

Ground-glass opacities are the most common imaging findings in COVID-19 in medical literature [11]. This is similar to the findings in the present study, in which a greater extent was observed in participants in Group A, suggesting a more severe lung injury with possible systemic involvement.

Thickened intralobular septa generally suggest lymphocytic infiltration. Therefore, due to direct lung injury in COVID-19, interstitial lung edema usually indicates an increase in pulmonary vascular permeability that results in the movement of fluid to the interstitial compartments [12]. However, this may also be associated with myocardial injury, since septal thickening may be induced by a hydrostatic component caused by vasoconstriction, myocardial ischemia, infarction, and arrhythmias [13]. A higher incidence of thickened intralobular septa was found in participants in Group A; therefore, the hypothesis of a myocardial injury should be considered and further investigated in COVID-19 patients.

Fibroatelectatic lesions, which increase the vascular resistance due to lung fibrosis [14], were more prevalent and more severe in group A, with an important role in the pathogenesis of cardiac pump dysfunction and secondary myocardial injury. In the lung zones with atelectatic changes, the gas exchange tends to be zero, with preservation of vascular perfusion and the appearance of right to left intrapulmonary vascular shunt. Thus, pulmonary hematosis decreases, worsening the hypoxia induced by the initial alveolo-interstitial injury. The hypoxic state and inflammation may play a key role in the pathogenesis of acute myocardial injury in COVID-19 [15].

The presence of mediastinal adenopathy was more prevalent among Group A participants. Therefore, we hypothesize that mediastinal adenopathy may follow extensive lung damageand may reveal bacterial suprainfection or an excessive inflammatory response. The accumulation of lymphocytes in the coronary microcirculation may increase coronary resistance to flow, trigger coronary vasospasm, and induce cardiomyocyte necrosis [16].

The prevalence of pericardial effusion among COVID19 patients has been reported to vary between 4.6% and 90.7% in critically ill patients and was reported as an incidental finding on CT scans; pericardial effusion found in severe cases of COVID-19 may be associated with myocardial dysfunction and an increase in overall mortality by 60% compared to cases without pericardial involvement [17]. From this study, pericardial effusion showed a similar prevalence in both Groups A and B (22% and 20%, respectively).

Tissue damage can generally be indicated by elevated levels of cytolytic enzymes, including LDH, and although lacking cardiac specificity, LDH has been shown in our current study to correlate with myocardial injury. Recent studies have indicated a higher risk of negative clinical prognosis and higher mortality in patients with elevated LDH, indicating myocardial injury by a general inflammatory response with premature cell death, and transitory or definitive cardiac cell ischemia due to thrombus formation, mainlyin conditions involving endothelial dysfunction [18,19]. Although not a specific marker for cardiac injury, increased levels of LDL may be detected in COVID-19 patients with a more severe form of pneumonia;in patients with high values of LDH, myocardial injury should also be considered as a differential diagnosis.

Some studies have shown the importance of D-dimers in the diagnosis of myocardial injury and acute coronary syndrome [20]. Similarly, our study also showed that D-dimers correlate with the risk of myocardial injury. Furthermore, multiple studies have shown that elevated D-dimer levels can predict poor prognosis and mortality in COVID-19 [21,22] because the endothelial dysfunction frequently seen in COVID-19 can result in thromboembolic events (stroke, myocardial infarction, renal or splenic infarction, etc.). The most cited pathophysiological mechanisms include endothelial dysfunction in massive inflammatory reponses [23], hypoxia-induced stimulation of thrombotic events [24], disseminated intravascular coagulation [25], invasive treatment, immobilization, and old age as a supplemental risk factor for thrombosis [26].

Although TnI and NT-pro-BNP are widely used as markersof myocardial injury, they are not fully specific. Therefore, additional markers such as serum valosin-containing protein (VCP), lectin mannose-binding 2 (LMAN2), and calpain-1 catalytic subunit (CAPN-1) should be used if available. These show a diagnostic specificity above 60% compared to CK-Mb (43.4%) and TnI (34.7%) [27,28].

Various types of viruses including Coxsackie B, enterovirus, HIV, parvovirus, and herpesvirus have been found to show cardiac involvement. After entering into cardiomyocytes and activating the innate immunity, the virus replicates and activates the acquired immune response, followed by disease resolution or further complications manifesting as dilated cardiomyopathy (enteroviruses), cardiomyocytes necrosis (Coxsackie B), and compromise of the systolic function. Not all myocardial injuries are exerted directly, for instance, parvovirus B endothelial damage may occur with diminished myocardial perfusion and ventricular dysfunction [16].

In HIV infection, the involvement of the myocardium is usually multifactorial—usually by a direct invasion of cardiomyocytes by the virus. However, HIV infection may be associated with other viral infections, drug-related cardiac toxicity, nutritional deficiencies, and prolonged immunosuppression [29]. Chronic immune activation and a persistent inflammatory state, with increased blood levels of the inflammatory cytokines—TNFα, —IL-6, and CRP [30] may induce cell apoptosis with secondary endothelial and myocardial dysfunction.

In a study conducted on 106 autopsied hearts Matsumori et al. [31] identified the presence of HCV infection in 13 hearts (21.3%) with myopathic disorders: in four hearts (33.3%) with myocarditis, in three hearts (11.5%) with dilated cardiomyopathy, and in six hearts (26.0%) with hypertrophic cardiomyopathy; myocarditis has also been reported in association with hepatitis A [32] and E [33]. Although the mechanism of cardiomyocyte injury in hepatitis is yet unknown, immune response to myocyte infiltration of the virus [10] should be considered.Aninflammatory response with increased release of cytokines is frequently found in hepatitis [34], which may also contribute to cardiac involvement.

COVID-19 myocarditis seems to have distinct inflammatory characteristics that distinguish it from other viral etiologies, with a wide variety of presentations ranging from dyspnea and chest pain to acute heart failure and possibly death [35]. The main mechanisms of myocardial injury in COVID-19 that have been reported include hyperinflammation and cytokine-mediated injury, direct myocardial invasion by the virus, the imbalance of oxygen supply and demand in conditions of respiratory failure and hypoxemia, hypercoagulability and the presence of endothelitis, and the impairement of coronary microvascular flow due to thrombi formation or plaque rupture with myocardial ischemia [36]. From a study, Guo et al.reported that approximately 28% of patients with COVID-19 developed myocardial injury that was diagnosed based on increased plasma level of troponin T [4]. Furthermore, secondary to mRNA SARS-CoV-2 vaccination, myocarditis and pericarditis may occur within a few days of vaccination, particularly after the second dose [37].

The administration of steroids is considered beneficial in the management of COVID-19 [38], because it reduces mortality by a third in patients with mechanical ventilation and by a fifth in those who require oxygen therapy [39]. Violi et al. showed that steroid treatment could reduce myocardial injury during the acute phase of community-acquired pneumonia and, eventually, cardiovascular events and mortality during a short- and long-term follow-up for patients [40]. However, in critically ill patients with prolonged immobilization and steroid administration, osteopenia or osteoporosis may occur and be worsened by eventual comorbidities [41,42].

*Study limitations*: No cardiac ultrasound was available for use in the management of patients on admission at the hospital, meanwhile, more cardiac measures such as MRI and echocardiography may be helpful for comprehensively estimating the extent of myocardial injury. Since cTnI and NT-pro-BNP may not be fully specific or sensitive for diagnosing myocardial injury, additional markers (such as VCP or endoplasmic reticulum stress-related secretory proteins) should be used if available.The results in this study are descriptive and have no prospective observation period; therefore, the obtained results need to be validated by further prospective studies and confirmed on larger samples. The present study only assessed the value of clinical, biochemical and imaging factors in determining the severity of myocardial involvement, without demonstrating specific pathways for cardiac injury.

## 5. Conclusions

This study showed that myocardial injury occurred in 12% of patients in the study group, and that routine lung sectional imaging along with non-specific biomarkers (LDH, D-dimers, CRP), can provide further value in the characterization of the disease burden, including myocardial injury, thus impacting patient care.

## Figures and Tables

**Table 1:** Comorbidities, medication and lifestyle characteristics in COVID-19 patients with and without myocardial injury.

Comorbidity		Group A (n, %)	Group B (n, %)
Overweight (BMI = 25–29.9 kg/m^2^)		4 (22.2)	43 (32.5)
Obesity (BMI > 30 kg/m^2^)		3 (16.6)	19 (14.4)
Diabetes mellitus		2 (11.1)	13 (9.8)
Chronic obstructive pulmonary disease		1 (5.5)	12 (9.1)
Viral chronic hepatitis		1 (5.5)	7 (5.3)
History of neoplasia		1 (5.5)	5 (3.8)
History of stroke		1 (5.5)	6 (4.5)
Dementia		0 (0)	1 (0.7)
Peptic ulcer		1 (5.5)	3 (2.2)
Chronic heart diseases *		0 (0)	0 (0)
Prior myocardial infarction		0 (0)	0 (0)
Smoking status	Active smokers	6 (33.3)	34 (25.7)
	Ex-smokers	2 (11.1)	17 (12.9)
	Non-smokers	10 (55.6)	81 (61.4)
Dyslipidemia		9 (50)	73 (55.3)
Systemic arterial hypertension		6 (33.3%)	25 (18.9%)
Use of cardiac medication	ACEI/ARB	5 (33.3)	21 (15.9)
	diuretics	1 (5.5)	9 (6.8)
	calcium channel antagonists	2 (1.1)	16 (12.1)
	beta-blockers	1 (5.5)	3 (2.3)
	statines	4 (22.2)	21 (15.9)

* patients with chronic heart disease were excluded from the study population; ACEI = angiotensin-converting-enzyme inhibitors; ARB = angiotensin II receptors blockers.

**Table 2:** Clinical and laboratory characteristics in COVID-19 patients with and without myocardial injury.

Demographic, Clinical, and Biological Characteristics	Group A (Median, IQR, [Q1; Q3])	Group B (Median, IQR, [Q1; Q3])	*p*-Value	OR [95% CI]
Age (years)	58, 19 [48.5; 67.2]	54.5, 24 [43.2; 67]	0.53	1.01 [0.978; 1.043]
Hospitalization period (days)	11.5, 9 [10; 19]	11, 7 [7; 14]	0.25	1.039 [0.976; 1.106]
SpO_2_ (%)	92, 8 [88; 96]	94, 7 [90; 97]	0.25	0.937 [0.840; 1.046]
Heart rate (beats/min)	80.5, 18 [68.7; 87]	77, 24 [65; 89]	0.48	1.012 [0.978; 1.047]
Systolic blood pressure (mmHg)	130, 35 [107.5; 142.5]	125, 24 [112.7; 136.7]	0.59	1.007 [0.982; 1.033]
Diastolic blood pressure (mmHg)	70.5, 20 [60; 80]	76, 13 [70; 83]	0.49	0.986 [0.947; 1.027]
Respiratory rate (breaths/min)	28, 13 [20; 33]	24, 9 [21; 30]	0.46	1.026 [0.958; 1.100]
TnI (ng/mL)	0.07, 0.15 [0.05; 0.19]	0.03, 0 [0.03; 0.03]	<0.001	-
NT-proBNP (ng/L)	98, 1089 [60; 1149]	44, 114 [9; 123]	0.001	1.001 [1;1.002]
LDH (UI/L)	296.5, [258.7; 344.5]	253.5, 107 [200.7; 308]	0.03	1.006 [1; 1.011]
Myoglobin (ng/mL)	108.8, 86 [78.9; 130.2]	93, 65.5 [62.5; 128]	0.30	1.004 [0.997; 1.01]
CRP (mg/L)	25, 62.1 [10.6; 72.7]	8.2, 23.2 [3.1; 23.3]	0.03	1.012 [1.002; 1.022]
Fibrinogen (mg/dL)	409, 250 [296; 545.5]	379.2, 186.7 [309.2; 496]	0.55	1.001 [0.998; 1.004]
Ferritin (ng/mL)	600.1, 761.7 [393.9; 1155.6]	276.3, 570.1 [118.9; 689]	0.07	1.001 [1; 1.002]
IL-6 (pg/mL)	28.7, 137.8 [3.6; 141.4]	8.5, 48.7 [1.4; 50.1]	0.24	1.001 [0.999; 1.002]
TNFα (pg/mL)	1.9, 12.4 [0; 12.5]	1.9, 7.3 [0; 7.3]	0.95	0.999 [0.979; 1.02]
D-dimers (ng/mL)	272, 691 [221; 912]	152, 120 [98; 217.5]	0.005	1.002 [1; 1.003]

SpO_2_—oxygen saturation; TnI—Troponin I; NT-proBNP—ventricular natriuretic peptide; CK-MB—creatine kinase muscle brain (isoenzyme); LDH—Lactate dehydrogenase; CRP—C reactive protein; IL-6—interleukin 6; TNFα—tumor necrosis factor α.

**Table 3:** Radiological characteristics in COVID-19 patients with and without myocardial injury.

Imaging Characteristics		Group A (n, %)/Median, IQR [Q1; Q3]	Group B (n, %)/Median [Q1; Q3]	*p*-Value	OR [95% CI]
Severity of lung disease	0	0 (0)	18 (13.6)	0.01	2.187 [1.128; 4.239]
	1	3 (16.6)	27 (20.4)		
	2	7 (38.8)	61 (46.2)		
	3	8 (44.4)	26 (19.7)		
Number of pulmonary segments with interstitial pneumonia		10.5, 7 [6; 13.2]	7, 7 [4; 11]	0.06	1.102 [0.994; 1.222]
Number of pulmonary segments with alveolar consolidation		0.5, 4 [0; 4]	0, 0 [0; 0]	0.13	1.206 [0.959; 1.516]
Number of patients with alveolar consolidation		9 (50)	26 (19.7)		
Number of pulmonary lobes with pneumonia	0	0 (0)	18 (13.6)	0.04	1.618 [1.029; 2.545]
	1	0 (0)	7 (5.3)		
	2	0 (0)	9 (6.8)		
	3	1 (5.5)	6 (4.5)		
	4	1 (5.5)	15 (11.3)		
	5	16 (88.8)	77 (58.3)		
Type of lung involvement	1	0 (0)	27 (20.4)	0.006	
	2	18 (100)	105 (79.6)		
Crazy paving pattern	1	1 (5.5)	59 (44.7)	0.02	2.255 [1.135; 4.479]
	2	13 (72.2)	50 (37.8)		
	3	4 (22.2)	23 (17.4)		
Vascular ectasia	0	9 (50)	88 (66.6)	0.19	1.519 [0.826; 2.793]
	1	5 (27.7)	26 (19.7)		
	2	4 (22.2)	18 (13.6)		
Extent of fibroatelectasis lesions	0	1 (5.5)	63 (47.7)	0.002	2.440 [1.372; 4.337]
	1	10 (55.5)	37 (28)		
	2	4 (22.2)	30 (22.7)		
	3	3 (16.6)	2 (1.5)		
Mediastinal adenopathies		10 (55.5)	40 (30.3)	0.04	2.875 [1.057; 7.823]
Pleural effusion		3 (16.6)	7 (5.3)	0.11	3.571 [0.834; 15.297]
Pericardial effusion		4 (22.2)	26 (19.7)	0.80	1.165 [0.354; 3.833]

**Table 4:** Correlations of clinical, laboratory, and imaging parameters with myocardial injury.

Clinical, Laboratory, and Imaging Characteristics	Spearman’s Rho	*p*-Value
Age (years)	0.04	0.63
SpO_2_ (%)	−0.10	0.20
Heart rate (beats/min)	0.05	0.56
Systolic blood pressure (mmHg)	0.03	0.71
Diastolic blood pressure (mmHg)	−0.12	0.119
Respiratory rate (breaths/min)	0.07	0.41
Hospitalization period (days)	0.12	0.13
NT-proBNP (ng/L)	0.265	0.004
LDH (UI/L)	0.190	0.02
Myoglobin (ng/mL)	0.10	0.27
CRP (mg/L)	0.209	0.01
Fibrinogen (mg/dL)	0.04	0.65
Serrum ferritin (ng/mL)	0.200	0.06
IL-6 (pg/mL)	0.120	0.28
TNFα (pg/mL)	−0.004	0.97
D-dimers (ng/mL)	0.322	0.001
Severity of lung disease	0.195	0.017
Number of pulmonary segments with interstitial pneumonia	0.149	0.06
Number of pulmonary segments with alveolar consolidation	0.218	0.007
Number of pulmonary lobes with pneumonia	0.214	0.009
Crazy paving pattern	0.213	0.009
Vascular ectasia areas	0.115	0.16
Type of lung involvement	0.173	0.03
Extent of fibroatelectasis lesions	0.257	0.002
Pleural effusion	0.148	0.07
Pericardial effusion	0.021	0.80
Mediastinaladenopathies	0.174	0.03

**Table 5:** Multivariable logistic regression model for patients with myocardial injury.

Regression Model Type	B	S.E.	Wald	*p*	OR	95% CI for OR	
						Lower	Upper
LDH	0.004	0.003	1.762	0.18	1.004	0.998	1.011
D-dimers	0.001	0.001	3.568	0.05	1.001	1	1.002
Extent of fibroatelectasis lesions	0.881	0.403	4.793	0.02	2.414	1.097	5.313
Mediastinal adenopathies	1.496	0.685	4.778	0.02	4.466	1.167	17.087
Constant	−5.288	1.255	17.761	0	0.005		

## Data Availability

The data presented in this study are available on request from the corresponding authors.
